# Social behavior and the microbiome

**DOI:** 10.7554/eLife.07322

**Published:** 2015-03-31

**Authors:** Jack A Gilbert

**Affiliations:** Department of Biosciences and Institute for Genomic and Systems Biology, Argonne National Laboratory, Chicago, United States and Department of Ecology and Evolution, and Department of Surgery, University of Chicago, Chicago, United Statesgilbertjack@anl.gov

**Keywords:** *Papio cynocephalus*, social behavior, gut microbiome, metagenomics, transmission, social network, other

## Abstract

Social interactions influence the communities of microbes that live in wild baboons.

**Related research article** Tung J, Barreiro LB, Burns MB, Grenier JC, Lynch J, Grieneisen LE, Altmann J, Alberts SC, Blekhman R, Archie EA. 2015. Social networks predict gut microbiome composition in wild baboons. *eLife* **4**:e05224. doi: 10.7554/eLife.05224

**Image** Baboons share microbes through social grooming. Image credit: Noah Snyder-Mackler

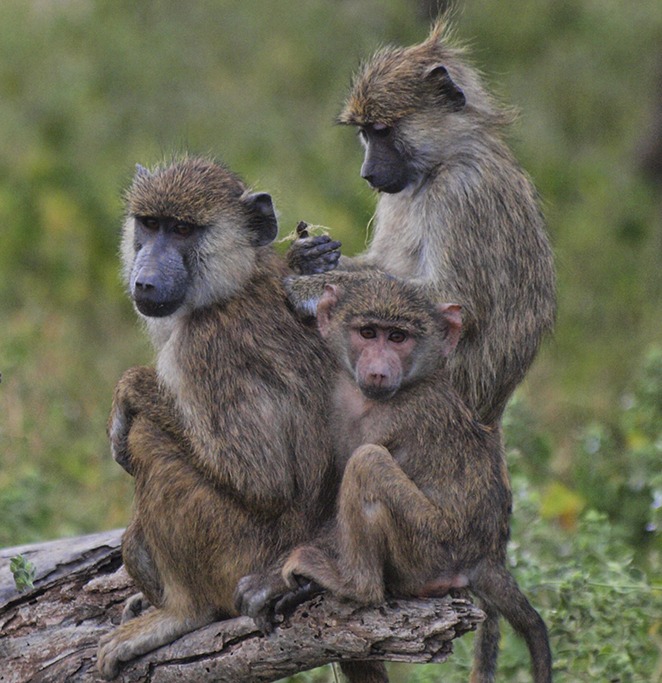

Why did social interactions evolve? Complex rules govern how we interact when we meet, and while these principles are often bewildering, they are also somehow intrinsic to the fabric of any human society. Now, in eLife, Elizabeth Archie, Jenny Tung and co-workers have provided compelling evidence to support the theory that physical interaction evolved in vertebrates to help share potentially health-promoting bacteria (Tung et al., 2015).

Tung et al. sequenced the genomes of the various microbes found in the faeces of 48 baboons from two different social groups in Amboseli in Kenya. They found that these ‘microbiomes’ differed between the two social groups, despite them living in overlapping areas and eating similar foods. It has been shown that diet influences the composition and structure of the microbial communities living in the human gut (David et al., 2013). So why were the microbiomes of these groups of baboons not more similar?

The answer lies in the fact that baboons interact by grooming each other (Figure 1). Baboons that groomed each other more frequently had more similar microbiomes, which suggests that physical interactions with others are very important in shaping the microbial communities in these individuals. Baboons don't tend to groom baboons from different social groups (much like humans), so social activities within a group act to consolidate shared communities of microbes.

**Figure 1.**
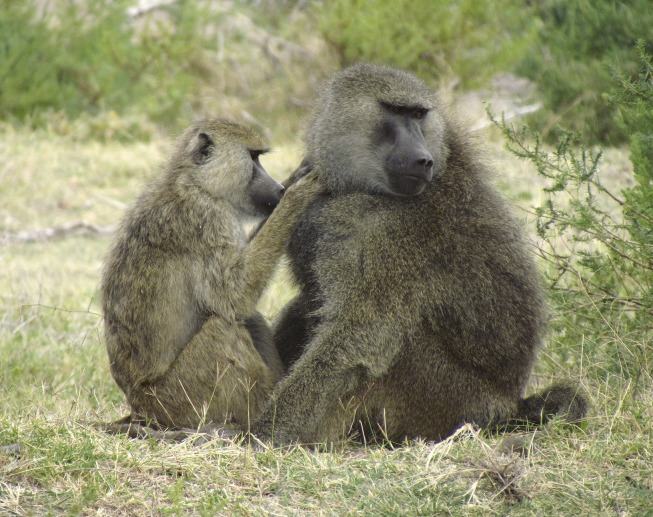
Baboons that groom each other more often have more similar gut microbiomes.

Image credit: Elizabeth A. Miller.

The need for physical interaction or the presence of others is central to human cognitive psychology. Embodied cognition is the study of how the presence of others affects thoughts, feelings, and behaviors (Meier et al., 2012); for example, we tend to distance ourselves physically from those we don't like or trust, whereas we tend to be more physically close and have more physical interactions with our friends and family.

It is highly probable that evolution has shaped social behavior. Physical interactions with those we love feels good; hugs, kisses, and even basic skin-to-skin contact have positive influences on our thoughts and feelings. The hand-shake—which is the standard mode of introduction in many societies—represents this concept. But why did these interactions evolve? Are they purely social constructs, or is physical interaction instinctive, driven by some selective advantage? Sharing microbes in this way can be beneficial: for example, bumblebees living in the same hive share bacteria through fecal pellets (Koch and Schmid-Hempel, 2011). This protects the bees from the virulent parasite *Crithidia bombi*, and therefore gives these bees a selective advantage over other bees that don't share bacteria.

Acquiring a beneficial microbiome may actually have shaped the evolution of the immune system, whose primary role is to maintain the balance of ‘good’ and ‘bad’ microbes in the microbiome. The immune system aims to control the exposure of host tissues to the microbiome, which it does in part by directly interacting with the bacteria. For example, the protein immunoglobulin A is released into the gut, where it binds to certain species of bacteria: this reduces the ability of the bacteria to move and keeps them away from the cells of the intestine (Hooper et al., 2012). It is likely that this mechanism is also used to capture certain beneficial microbes and retain them in the gut.

Sharing of microbes through physical interaction has been shown to happen in several vertebrate species. For example, my own work in humans demonstrates that family relationships and co-housing can influence the similarity of the microbiomes of individuals (Lax et al., 2014). In fact, in one of the groups examined, a young couple was shown to have much more similar microbiomes on their skin and in their nose than a lodger living with them in their home: however, the lodger had more microbes in common with the couple than he did with anyone else in the study.

Unlike the baboons—who only share significant amounts of microbes due to physical grooming activity—humans can also share microbes through the artificial environment we have constructed for ourselves. Over the last 100 years or so, these indoor environments have become increasingly isolated from the natural world outside. The microbiome of individuals living in the same indoor space can be shared through the air and via surfaces because humans are the main source of the microbes, and therefore most of the microbes in the space are readily able to colonize the human occupants.

This sharing of microbes might seem like a good idea; it worked for the bees. However, there is now mounting evidence to suggest that the over-sharing of the microbiome may be reducing our exposure to richer microbiomes from other sources, thereby limiting the development of our immune system (Lax et al., 2015). Wild baboons are exposed to many different sources of microbes in their environment, and—while their physical interactions may indeed help to share beneficial bacteria—these other microbes are also likely to support their physical, immunological and neurological development.

One of my colleagues, the eminent microbiologist and microbial ecologist Norman Pace, says that his exploration of the microbial world has made him reluctant to share other people's microbiomes (Personal Communication). In fact, Norman now only ‘fist bumps’ when he meets other people, and never shakes their hands. Is this reduced microbial exposure potentially detrimental to his health? Or could it be that in our modern world, Norman has the right idea?
